# Editorial: Next generation sequencing: applications in foodborne pathogens

**DOI:** 10.3389/fmicb.2024.1415863

**Published:** 2024-05-01

**Authors:** Alexandre Lamas, Alejandro Garrido-Maestu

**Affiliations:** ^1^Food Hygiene, Inspection and Control Laboratory (Lhica), Department of Analytical Chemistry, Nutrition, and Bromatology, Veterinary School, Universidade de Santiago de Compostela (USC), Lugo, Spain; ^2^Laboratory of Microbiology and Technology of Marine Products (MicroTEC), Instituto de Investigaciones Marinas (IIM), CSIC, Vigo, Spain; ^3^International Iberian Nanotechnology Laboratory, Av. Mestre José Veiga s/n, Braga, Portugal

**Keywords:** next generation sequence (NGS), long read sequence technology, short-read sequencing, bioinformatics, foodborne pathogen, food safety

While the complete genome of a bacterium, a strain of *Haemophilus influenzae*, was sequenced for the first time in the early 1990s, the routine introduction of sequencing techniques into food safety laboratories was still a pipe dream. Although with the development of Sanger sequencing, genomic characterization studies of foodborne pathogens increased, it was not until the second decade of the 21st century, when sequencing technologies revolutionized the field of food safety with the appearance of massive sequencing. One of the most exploited applications was Whole Genome Sequencing (WGS), which allowed progress from phenotypic to genotypic characterization of bacteria. This technique allowed for virulence, and resistance, genomic characterization, as well as to determine phylogenetic relationship between isolates, particularly useful in outbreak investigations. In parallel to this technology, a whole range of bioinformatic tools have been developed. Furthermore, in recent years the use of bioinformatic software has been democratized with the appearance of web-based platforms that allow these programs to be used by researchers with limited bioinformatics kills. This Research Topic compiles articles that demonstrate the potential of massive sequencing in food safety, beyond the “classical” applications.

Brown et al. sequenced 88 *Listeria monocytogenes* isolates from five dairy processing industries collected between 2007 and 2017 in the same region. The authors performed multi-locus sequence type (MLST), clonal complex, core genome MLST (cgMLST), *sigB* allelic profile, and whole genome SNP analysis. The results showed 11 cgMLST types, and that most of the strains belonged to lineage II, one of the most common in foods. The distribution of virulence genes varied among different isolates and farms. But the most interesting thing is that 60 strains of the 72 isolated from the same facility in different years presented high similarity (ranging from 0 to 16 SNPs), which gives an idea of the persistence capacity of *L. monocytogenes* strains in the environment. a food industry Also focused on *L. monocytogenes*, Jeong et al. carried out a complete study in a chicken slaughterhouse comprising a sampling period of 3 years (2019–2021). In this study, samples were taken at different points of the slaughterhouse to identify contamination routes and the presence of *L. monocytogenes* was determined by culture-dependent and independent methods. By using 16s RNA amplicon sequencing it was possible to trace the main sources of contamination of chicken carcasses during slaughter, especially important to determine the critical points that need special monitoring.

Zhong et al. performed WGS of 186 *Clostridium perfringens* isolates collected in China between 2013 and 2021 from humans, animals, and food. The results showed great genetic diversity in the isolates with 135 different sequence types (ST) and no connection between host, geographic distribution or toxinotype was found in the analyzed isolates. The researchers also observeda high prevalence of tetracycline resistance genes. These studies were of great value to elucidate the phylogenetic relationship between strains distributed in a country over the years, and locate particularly dangerous lineages.

WGS data deposited in databases are also very useful for understanding the behavior of foodborne pathogens. Mao et al. analyzed the *sigB* factors of 46,921 *L. monocytogenes* genomes available in the NCBI database. The researchers found two main protein variants, SigB type I and SigB type II. Furthermore, SigB type I variant was mainly correlated with lineages I and III and SigB type II was mainly related to lineage II. Complementation with phenotypic studies demonstrated a greater capacity of SigB type I to promote cell invasion, cytotoxicity, promote biofilm formation and cold tolerance. These types of studies demonstrate the importance of analyzing genomic data from strains isolated throughout the world to understand factors related to the pathogenesis of foodborne pathogens.

The development of long read sequencing by Pacific Biosciences, aka PacBio, and Oxford Nanopore Technologies (ONT) have revolutionized this research field. Specially ONT has allowed massive sequencing to be used routinely in food safety laboratories both for identification and characterization of pathogens due to the low initial investment necessary to set up this technology in laboratories through the use of MinION equipment. Several articles of the present Research Topic focused in the application of this specific technology.

Lamas et al. selected the single use ONT Flongle flow cells, in combination with the web-based platform Galaxy, for rapid serotyping of *Salmonella* isolates, and the characterization of genomic antimicrobial resistance and virulence. This article describes a step by step protocol for the sequencing and bioinformatic analysis of *Salmonella* isolates so that it can be applied in laboratories with low resources, and with limited bioinformatic skills.

Another approach is based on the use of metagenomics. Maguire et al. employed this approach for the detection of Shiga toxin-producing *Escherichia coli* in agricultural water including a short enrichment step prior to DNA isolation from the sample. They performed metagenomics with short Illumina reads alone or in combination with long ONT reads, and determined which was the best option to characterize the strains isolated, and to stablish a phylogeny in the event of an outbreak. The results showed a limit of detection between 10^5^ and 10^7^ CFU/mL, and that it was possible with at least 10^7^ CFU/mL to obtain a complete fragmented genome using a hybrid assembly, with correct identification of serotype and virulence genes compared to the reference assembly. Buytaers et al. developed a different metagenomic approach. One of the limitations of metagenomics in the identification of pathogens in complex samples is the interference of DNA from other bacteria, and/or the host/food. If targeted sequencing is not performed, a large part of the sequenced DNA corresponds to that interfering genetic material, limiting the results obtained. To solve this problem, the researchers used nanopore adaptive sampling. This can be considered software-directed enrichment. Through the use of reference databases entered by the user, only those reads of interest are sequenced. With this strategy, the authors were able to identify, characterize, and perform phylogenetic analyses of *Staphylococcus aureus* present in samples of artificially contaminated mashed potato, without the need to perform prior enrichment. Furthermore, they demonstrated that this strategy was more effective than the use of extraction kits that eliminated host DNA.

## Author contributions

AL: Conceptualization, Writing – original draft. AG-M: Conceptualization, Writing – review & editing.

